# Expressive suppression moderates the relationship between PTSD from COVID-19 and somatization and validation of the Arabic version of Patient Health Questionnaire-15 (PHQ-15)

**DOI:** 10.1371/journal.pone.0293081

**Published:** 2024-01-25

**Authors:** Antonio Nehme, Sara Moussa, Feten Fekih-Romdhane, Ecem Yakın, Souheil Hallit, Sahar Obeid, Georges Haddad

**Affiliations:** 1 School of Medicine and Medical Sciences, Holy Spirit University of Kaslik, Jounieh, Lebanon; 2 Faculty of Medicine, University of Balamand, Koura, Lebanon; 3 The Tunisian Center of Early Intervention in Psychosis, Department of Psychiatry “Ibn Omrane”, Razi Hospital, Manouba, Tunisia; 4 Faculty of Medicine of Tunis, Tunis El Manar University, Tunis, Tunisia; 5 Centre d’Études et de Recherches en Psychopathologie et Psychologie de la Santé, Université de Toulouse-Jean Jaurès, UT2J, Toulouse, France; 6 Applied Science Research Center, Applied Science Private University, Amman, Jordan; 7 Research Department, Psychiatric Hospital of the Cross, Jal Eddib, Lebanon; 8 Social and Education Sciences Department, School of Arts and Sciences, Lebanese American University, Jbeil, Lebanon; McMaster University, CANADA

## Abstract

**Background:**

Lebanese adults have been crippled for years by several crises, including the lately COVID-19 pandemic. These massive civilian traumas have increased the risk of post-traumatic stress disorder (PTSD) in this population. Extensive literature pointed to the association between PTSD and somatization; however, the nature of this relationship remains unknown. We sought to contribute further to work in this area by testing the moderating role of emotion regulation in the relationship between COVID-19- related PTSD and somatization. As a secondary objective, we aimed to examine the psychometric properties of an Arabic translation of the somatization measure Patient Health Questionnaire-15 (PHQ-15) in terms of factorial validity and internal consistency before its use in the present study.

**Methods:**

This cross-sectional study was conducted between September and October 2021. A total of 403 Lebanese adults residing in Lebanon were recruited. Eligible participants received an online link to the survey. The Patient Health Questionnaire-15 was used to assess somatization, PTSD Checklist–Civilian Version for PTSD and Emotion Regulation Questionnaire for emotion regulation.

**Results:**

The results of the exploratory factor analysis (EFA) revealed a three-factor solution explaining 48.79% of the common variance. Confirmatory Factor Analysis results of the three-factor model obtained in the EFA indicated a good fit with a significant CFI of 0.98, TLI 0.98 and a GFI of .97, a RMSEA of .04 [90% CI .01, .06]. Higher PTSD symptoms were associated with somatization. In addition, we found that one specific ER component, i.e. expressive suppression, significantly moderated the relationship between PTSD from the COVID pandemic and somatization. In particular, the interaction PTSD from the COVID-19 pandemic by expressive suppression was significantly associated with somatization; at low, medium and high levels of expressive suppression, higher PTSD from the COVID-19 pandemic was significantly associated with higher somatization scores. As for our secondary objective, findings revealed that the Arabic version of the PHQ-15 exhibited good psychometric properties. In particular, the scale yielded a three-factor structure, and good internal consistency (Cronbach’s alpha = 0.87).

**Conclusion:**

The moderating role of expressive suppression on the link between PTSD and somatization presents a novel finding in the field of trauma. Additionally, making a psychometrically sound Arabic version of the PHQ-15 available is a valuable addition to the literature.

## Background

The coronavirus disease, caused by the severe acute respiratory syndrome coronavirus 2 (SARS-CoV-2) virus, is thought to be responsible of one of the deadliest pandemics in the contemporary history. As of March 15, 2021, Lebanon counted up to 418,448 confirmed cases and 5380 deaths [1]. Worldwide, by June 8 2022, there have been 530,896,347 confirmed cases of COVID-19, including 6,301,020 deaths reported to the World Health Organization (WHO) [2]. This fast spreading pandemic has obliged governments to adopt unfamiliar precautionary measures; in Lebanon, nationwide lockdowns have been applied, social gatherings were banned, social distancing was encouraged, people were obliged to wear facemasks in public, and at the end a vaccine become available [3]. In this context, a Lebanese study [4] conducted between November and December 2020 revealed that less than a quarter of Lebanese adults expressed willingness to accept the COVID-19 vaccine when it becomes available, a rate far below those of the UK (64%) [5] and the US (57.6%) [6]. All these measures required millions of peoples to suddenly change their routine and habits, what lead to severe psychological consequences in both infected and non-infected individuals [7]. Particularly, and according to prior studies on previous coronavirus outbreaks, COVID-19 was expected to be associated with a high likelihood of developing post-traumatic stress disorder (PTSD) symptoms [8]. PTSD is a condition that can develop in individuals who experienced or witnessed a scary, shocking, terrifying, or dangerous past event [9]. Experiences from previous epidemics have shown that 42% of MERS survivors and 26% of SARS survivors presented PTSD symptoms following the outbreak [10, 11]. Similarly, extensive body of evidence from systematic reviews and metaanalyses reported high prevalence estimates of COVID-19-related PTSD in various populations worldwide, including the adult general population [12],children and adolescents [13] university students [14, 15], teachers [16], healthcare workers [17, 18], survivors of COVID-19 infection [19], and in high risk groups (e.g., older adults, pregnant women, obese individuals, smokers, individuals with obesity or other comorbidities) [20]. Arab people are of no exception (e.g., [21–24], even though evidence on the topic from this population remains relatively scant.

The situation in Lebanon may have even been among the worst, as the country was already weakened by the political and economic instability it has been going through over the last years (e.g., the 1975–1990 civil war, the 1985–2000 Israeli occupation of Southern Lebanon, the 2005–2021 repetitive assassinations and bombings). The COVID-19 sanitary crisis has thus exacerbated the social and financial unrest in the country. Households experienced more salary cuts, job losses, unemployment, and mortgage payment difficulties [25–27]. The already fragile healthcare system was rapidly overwhelmed and exhausted because of the scarce resources, including medications and medical supply shortages [27–30]. Exposure to all the above-mentioned chronic, repeated and highly traumatic events have put millions of Lebanese at an increased risk for developing complex PTSD symptoms [31]. In this regard, some empirical evidence pointed to PTSD prevalence rates of 31.5% from COVID-19, among Lebanese university students during May-August 2021 [32]. PTSD itself has been shown to increase the likelihood of poor general health and reporting of severe somatic symptoms [33, 34]. In the next session, we will take an in-depth look at the relationship between PTSD and somatization.

### The relationship PTSD-somatization

Somatization is when an individual exhibits multisystem physical symptoms with no physical cause identified [35, 36]. These symptoms are highly debilitating, as they often pose a vicious cycle of pain and distress [35, 36], and are associated with excessive thoughts, feelings and conducts that can lead to tremendous disability and wretchedness [37]. One of the widely used measures of the somatization construct is the Patient Health Questionnaire-15 (PHQ-15) [38], which is comprised of 15 items involving different somatic symptoms helping to detect individuals at risk for somatoform disorders. Somatization is suggested to be an adaptive response to psychological distress [39]. Several previous studies pointed to a close link between PTSD and excess of somatization [40], and to newly developed somatic symptoms in people with PTSD [36], Disasters, such as the COVID-19 pandemic, carry a risk of both PTSD and somatization to directly and indirectly affected people [41, 42]. Specifically, PTSD is found to be associated with a range of somatic problems (e.g., unexplained tinnitus, blurry vision, dizziness), and multiple medical conditions (e.g., respiratory, cardiovascular, and gastrointestinal disorders) [33]. More broadly, a meta-analytic review found that individuals with high PTSD symptoms displayed significantly more general medical conditions, greater general health symptoms, higher frequency and severity of pain, cardio-respiratory symptoms, and gastrointestinal complaints [43]. PTSD and somatization are severe mental health conditions that adversely impact quality of life [43]. Therefore, if PTSD is accompanied by post-traumatic somatization, then it is highly relevant to evaluate such symptomatology for this population and plan treatment accordingly. Hence the major importance of investigating the relationship between PTSD and somatization, and exploring potential factors influencing this relationship. As one of the factors, emotion regulation (ER) is assumed to relate to post-traumatic responses. In the present study, we propose to examine the role of ER as moderator in the relationship PTSD-somatization.

### The moderating effect of ER on the relationship between PTSD and somatization

According to John and Gross [44], ER refers to “the process by which individuals influence what emotions they have, when they have them, and how they experience and express those emotions”. Several studies have observed the pivotal role of ER difficulties in PTSD [45, 46]. Hence, ER implicates managing different systems, including physiological arousal, facial expressions, actions, incentive, and cognitive evaluations to modify or sustain an emotional state [47]. It can be broken down into different components essentially, namely self-awareness, cognitive reappraisal, adaptability, and emotion suppression. There is sufficient evidence that ER is related to PTSD symptoms [48]. Research suggested that individuals experiencing difficulties in regulating their emotions seem to be motivated to rely on avoidant coping strategies to regulate their PTSD symptoms what will ultimately lead to exacerbated conditions as patients never learns corrective information [48, 49]. Additionally, previous studies showed that difficulties in ER are not only associated with the severity of PTSD symptoms in a civilian sample [50] they also seem to play an important role in the chronicization of PTSD in civilians [51]. Other studies have shown positive effects for acceptance and cognitive reappraisal in a sample of veterans [52] and an effective treatment of PTSD can also reduce ER difficulties [53]. In addition to its association with somatoform disorders, ER is thought to be closely associated with somatic symptoms. A systematic review that assessed the role of ER on chronic pain, included 15 studies and concluded that maladaptive ER may be an important risk factor in the development and maintenance of chronic pain [54]. Another study suggested a potential association between emotion dysregulation and Psychogenic non-epileptic seizures (PNESs) [55]. Expressive suppression of emotions in particular was highly associated with somatization [56–58] and patients who suppress their emotions tend to suffer more from the impact of somatic symptoms [59]. Similarly, a study showed that patients with somatic symptom disorders had a lower capacity to nonverbally express their feelings than controls [60]. Based on its association with somatization on one hand and PTSD on the other, ER could serve as a moderator on the link between these two conditions.

### Rationale of the present study

The main motive of the present study was to advance our understanding of the yet unknown and under-researched association between PTSD and somatization, through an examination of possible moderating mechanisms. We believe that exploring, for the first time, the moderating role of ER in the path from PTSD to somatization is crucial for preventing and alleviating PTSD and somatization among individuals exposed to trauma. If ER is established as a moderator, it can be useful for developing and implementing prevention and intervention strategies considering the role of ER in attenuating the connection between PTSD and somatization. We also sought to contribute to the literature by investigating a highly vulnerable, but largely under-studied, population from Lebanon. There is some evidence that the prevalence of somatic symptoms related to PTSD and the way how trauma-caused symptoms are interpreted and manifest showed wide cross-cultural variability [61]. Therefore, as a country that is constantly tormented by a series of traumas and crises, Lebanon may provide fertile ground for development of PTSD and somatic disorders [40, 62]. Additionally, it is important to test our hypotheses in Lebanon where mental health is not a priority and facing major difficulties [25], and where mental illnesses are underestimated and stigmatized [63]. Therefore, the objectives of this study were to examine the association between PTSD symptomatology, particularly from the COVID-19 pandemic, and somatization, in addition to the moderating effect of emotion regulation in these associations. As a secondary objective, we aimed to examine the psychometric properties of an Arabic translation of the PHQ-15 in terms of factorial validity and internal consistency before its use in the present study.

## Methods

### Ethics approval and consent to participate

The study was approved by the Ethics Committee at the Psychiatric Hospital of the Cross (HPC-040-2021). All participants gave their informed written consent when submitting the online form. International guidelines were followed for the study.

### Study design

This cross-sectional study was conducted between September and October 2021. A total of 403 participants were enrolled from all Lebanese Mohafazat. Individuals above 18 years old and residents of Lebanon were allowed to participate. The sample was collected through a snowball technique (convenient sampling) via social applications (WhatsApp, Instagram), where the research team initiated the contact with people, who were consequently asked to forward the link to other friends and family members. Inclusion criteria for participation included being of a resident and citizen of Lebanon of adult age (aged ≥ 18 years). Excluded were those who refused to fill out the questionnaire. No exclusion criteria were applied. No monetary compensation was given for participating in the study.

### Sample size calculation

Following the recommendations of Comrey and Lee [64], a minimum sample of 10 participants per scale’s item are needed to conduct an exploratory factor analysis. Since the PHQ-15 scale is composed of 15 items, a minimal sample of 150 participants was needed for the exploratory factor analysis (EFA). For the confirmatory factor analysis (CFA), the minimum sample size ranges from 3 to 20 times the number of the scale’s variables [65], therefore, we assumed a minimum sample of 45–300 participants needed to have enough statistical power.

Using the multivariate regression option (deviation from zero), considering an effect size f^2^ = 2%, a type I error of 5%, a type II error of 20%, and 10 variables to be included in the final model, the G-power software [66] estimated a minimal sample of 395.

### Questionnaire

The Arabic language native to Lebanon was used in our anonymous questionnaire. The initial part assessed the sociodemographic characteristics (age, sex, marital status, educational level, and household crowding index). The household crowding index, reflecting the socioeconomic status of the family, was calculated by dividing the number of persons in the house by the number of rooms in the house excluding the bathrooms and kitchen [67]. In addition, participants were asked about their history of medical illnesses. The second piece of the questionnaire consists of different scales to assess each variable:

#### Patient Health Questionnaire-15 (PHQ-15)

PHQ-15 is a self-administered questionnaire composed of 15 items, each scored using a Likert scale with 0 “not bothered at all”, 1 “bothered a little”, 2 “bothered a lot” [68], used to assess if participants had experienced physical symptoms such as chest pain, gastrointestinal disturbance and trouble sleeping during the last month. Higher scores are associated with more severe somatization. Cutoff points of 5, 10 and 15 represent mild, moderate, and severe symptom levels [69] (In this study, Cronbach’s alpha = 0.87).

#### PTSD Checklist–Civilian Version (PCL-C)

The PTSD Checklist (PCL-C) is another self-report rating scale, comprises of 17 items for evaluating post-traumatic stress disorders (PTSD). Examinees show the amount they have been troubled by every manifestation in the previous month utilizing a 5-point scale [70]. (In this study, Cronbach’s alpha = 0.96 for PTSD from coronavirus).

*Emotion Regulation Questionnaire (ERQ)*. This ten-item questionnaire [71], validated in Lebanon [72], was used to assess if individuals regulate their emotions using cognitive reappraisal or expressive suppression. This scale is scored based on a 7 points Likert scale ranging from 1 (strongly disagree) to 7 (strongly agree). Higher scores indicate a larger dependence on the concerned strategy (in this study, Cronbach’s alpha = 0.89 for cognitive reappraisal and 0.79 for expressive suppression).

### Translation procedure

The translation procedure was done following international norms [73]. For the PHQ-15 and PCL-C, two survey independent translators were involved, the first made a translation from English into Arabic, and the second made the back-translation. The translators reviewed the two versions to identify irregularities and contrasts were settled with comprehension. A pilot study was done on 30 participants to make sure all questions are clear.

### Statistical analysis

The SPSS software v25 was used for data analysis. No missing data was found since questions were required. Reliability was checked using Cronbach’s alpha for the total scales and subscales. To check the psychometric properties of the PHQ-15, we employed an EFA-to-CFA [74] strategy using the RStudio (Version 1.4.1103 for Macintosh) the Lavaan and semTools packages. The original sample was randomly divided into two subsamples, one used for the EFA and the other for the CFA. The Kaiser-Meyer- Olkin (KMO) measure of sampling adequacy, and Bartlett’s test of sphericity was used to ensure the adequacy of the data [75]. In EFA, the number of factors underlying PHQ-15 items was determined on the basis of the screen test [76]. Based on the factors from the EFA, we conducted a Confirmatory Factor Analysis (CFA) using the data from the second subsample. Parameter estimates were obtained using the following fit indices: Comparative Fit Index (CFI), the Tucker-Lewis Index (TLI), Goodness of Fit Index (GFI), Standardized Root Mean Square Residual (SRMR) and Root Mean Square Error of Approximation (RMSEA). Values ≥0.90 for the GFI, CFI and TLI indicate a good model fit, values at or below 0.08 for the RMSEA, and 0.06 for the SRMR indicate good fit of the model to the data [77, 78].

The somatization score followed a normal distribution as shown by a skewness and kurtosis values between -2 and +2. The Student t-test was used to compare two means (e.g., somatization scores between gender groups), whereas the Pearson test correlated two continuous variables (e.g., somatization scores and age). A linear regression was done, taking the somatization score as the dependent variable. The Process Macro v3.4 model 1 (add-on for SPSS) [79] was used to conduct moderation models to examine the potential moderating effect of cognitive reappraisal/expressive suppression (moderators) on the relationship between PTSD from COVID-19 (independent variables) and somatization (dependent variable). Independent variables entered in the linear and moderation regressions were those that showed an effect size/correlation ≥ │0.24│ to achieve more parsimonious models [80]. The absence of multicollinearity was determined via the calculation of the Variance Inflator Factor (VIF < 5). R^2^ value was calculated to determine how much the independent variables entered in the model explained somatization. P < 0.05 was deemed statistically significant.

## Results

### Sociodemographic characteristics of the participants

A total of 403 enrolled in this study, with a mean age of 32.76 ± 13.24 years and 65.5% females. Other details are found in Table 1. Moreover, 133 (33.0%) and 113 (28.0%) had moderately severe and severe depression respectively.

**Table 1:** Sociodemographic characteristics of the participants (N = 403).

Variable	N (%)
**Sex**	
Males	139 (34.5%)
Females	264 (65.5%)
**Marital status**	
Single / divorced / widowed	266 (66.0%)
Married	137 (34.0%)
**Education level**	
Secondary or less	68 (16.9%)
University	335 (83.1%)
**Mouhafaza**	
Beirut	31 (7.7%)
Mount Lebanon	109 (27.0%)
North	255 (63.3%)
South	6 (1.5%)
Bekaa	2 (0.5%)
	**Mean ± SD**
Age (in years)	32.76 ± 13.24
Household crowding index	1.05 ± 0.51
Somatization	7.06 ± 5.22
PTSD from COVID-19 pandemic	36.78 ± 15.94
Cognitive reappraisal	22.69 ± 9.21
Expressive suppression	13.45 ± 6.01

### Exploratory and confirmatory factor analyses

Bartlett’s test of sphericity, χ2(105) = 1290.821, p < .0001, and KMO (.89) indicated that the PHQ-15 items were adequate for EFA. The results revealed a three-factor solution explaining 48.79% of the common variance (all item factor loadings ≥. 4) (Table 2). CFA of the three-factor model obtained in the EFA indicated a good fit with a significant CFI of 0.98, TLI 0.98 and a GFI of .97, a RMSEA of .04 [90% CI .01, .06].

**Table 2:** Items of the PHQ-15 scale in English and factor loadings derived from the Exploratory Factor Analyses (EFA) in the first subsample and standardized estimates of factor loadings from the Confirmatory Factor Analysis (CFA) in the second subsample.

Item	EFA			CFA		
	Factor 1	Factor 2	Factor 3	Factor 1	Factor 2	Factor 3
1.Stomach pain			.38			.64
2.Back pain	.57			.56		
3.Pain in your arms, legs, or joints.	.47			.50		
4.Menstrual cramps or other problems with your periods (women only)	.36			.57		
5.Headaches	.60			.64		
6.Chest pain		.52			.76	
7.Dizziness		.48			.83	
8.Fainting spells		.91			1.02	
9.Feeling your heart pound or race	.40			.76		
10.Shortness of breath		.49			.65	
11.Pain or problems during sexual intercourse		.75			.70	
12.Constipation, loose bowels, or diarrhea			.48			.67
13.Nausea, gas, or indigestion			.93			.88
14.Feeling tired or having low energy	.75			.88		
15.Trouble sleeping	.63			.72		

### Bivariate analysis

The bivariate analysis results are summarized in Tables 3 and 4. Higher number of comorbidities, and PTSD for the COVID pandemic were significantly associated with more somatization. Females scored significantly higher than males in terms of PTSD following the COVID-19 pandemic (38.93 ± 16.73 vs 32.71 ± 13.48; *t*(401) = -3.78; p < .001), anxiety (27.17 ± 8.38 vs 24.74 ± 8.07; *t*(401) = -2.80; p = .005), depression (19.42 ± 6.45 vs 17.18 ± 6.66; *t*(401) = -3.27; p = .001). Furthermore, they scored lower in terms of expressive suppression (12.92 ± 5.95 vs 14.44 ± 6.03; *t*(401) = 2.42; p = .016) compared to males. Finally, no significant difference was found between males and females in terms of cognitive reappraisal (22.99 ± 9.28 vs 22.53 ± 9.19; *t*(401) = .47; p = .638).

**Table 3:** Correlation matrix of continuous variables.

	1	2	3	4	5	6	7	8	9	10	11
1. Somatization	1										
2. Age	.01	1									
3. Household crowding index	.04	-.10	1								
4. Number of comorbidities	.30***	.23***	.14**	1							
5. Cognitive reappraisal	-.08	-.14**	-.05	-.11*	1						
6. Expressive suppression	-.03	-.16**	-.04	-.03	.56***	1					
8. PTSD from COVID-19 pandemic	.38***	-.07	.08	.18***	.11*	.12*	.77***	1			

*p<0.05

**p < .01

***p< .001; numbers in the table refer to correlation coefficients obtained from the Pearson test.

**Table 4:** Bivariate analysis of categorical variables associated with the somatization score.

Variable	Mean ± SD	*P*	Effect size
**Sex**		**< .001**	.876
Males	4.35 ± 4.32		
Females	8.49 ± 5.09		
**Marital status**		.627	.054
Single / divorced / widowed	7.15 ± 5.38		
Married	6.88 ± 4.91		
**Education level**		**.015**	.305
Secondary or less	8.46 ± 5.95		
University	6.78 ± 5.03		

Numbers in bold indicate significant p-values.

### Multivariable analysis

In the multivariable model taking sociodemographic variables and PTSD scores as independent variables, female gender (Beta = 3.36), a higher number of comorbidities (*Beta = 0*.*84*), higher PTSD from the COVID pandemic (Beta = 0.06) were significantly associated with more somatization (Table 5).

**Table 5:** Multivariable analysis: Linear regression (using the ENTER model) taking the somatization score as the dependent variable.

	Unstandardized Beta	Standardized Beta	*p*	95% CI	VIF
Sex (females vs males*)	3.36	.31	**< .001**	2.44; 4.28	1.039
Number of comorbidities	.84	.22	**< .001**	.52; 1.17	1.058
Education level (university vs secondary or less*)	-.98	-.07	0.098	-2.14; 0.18	1.025
PTSD from COVID pandemic	.06	.17	**0.010**	.01; 0.10	2.462

*Reference group; Numbers in bold indicate significant p-values. Nagelkerke R^2^ = .305

### Moderation analysis

The results of the moderation analysis (Table 6) were adjusted over the following confounding variables: sex, number of comorbidities, education and PTSD from COVID-19 pandemic. The interaction PTSD from the COVID pandemic by expressive suppression (Beta = .005; p = 0.031; 95% CI .001-.009) was significantly associated with somatization (Table 6, Model 1a); those variables explained 31.4% of the variance, *F*(7, 395) = 25.88, *p* < .001. At moderate (Beta = .05; p = .012) and high (Beta = .08; p = .001) levels of expressive suppression, higher PTSD from the COVID pandemic was significantly associated with higher somatization (Table 7). This effect was not seen at low levels of expressive suppression (Table 7).

**Table 6:** Moderation analyses.

	Beta	*t*	*p*	95% CI
**Model 1: PTSD from COVID-19 pandemic as the independent variable**				
**Model 1a: Cognitive reappraisal as the moderator**				
PTSD from COVID-19 pandemic	.04	.096	.338	-.04; .12
Cognitive reappraisal	-.08	-1.33	.184	-.19; .04
Interaction PTSD from COVID-19 pandemic by cognitive reappraisal	.001	.55	.584	-.002; .004
**Model 1b: Expressive suppression as the moderator**				
PTSD from COVID-19 pandemic	-.01	-.29	.775	-.08; .06
Expressive suppression	-.20	-2.26	**.024**	-.37; -.03
Interaction PTSD from COVID-19 pandemic by expressive suppression	.005	2.17	**.031**	.001; .009

Numbers in bold indicate significant moderation. R^2^ values for model 1a = .313; model 1b = .312; model 2a = .309; model 2b = .314

**Table 7:** Conditional effects of the focal predictor (PTSD from COVID-19 pandemic) at values of the moderator (expressive suppression).

Moderator	Beta	*t*	*p*	95% CI
Low (= 7.43)	.03	.97	.331	-.03; .08
Moderate (= 13.45)	.05	2.51	**.012**	.01; .10
High (= 19.46)	.08	3.33	**.001**	.03; .13

Numbers in bold indicate significant *p* values.

## Discussion

As expected, this study demonstrated that higher PTSD symptoms were associated with somatization. In addition, we found that one specific ER component, i.e. expressive suppression, significantly moderated the relationship between PTSD from the COVID pandemic and somatization. As for our secondary objective, findings revealed that the Arabic version of the PHQ-15 exhibited good psychometric properties. In particular, the scale yielded a three-factor structure, and good internal consistency (Cronbach’s alpha = 0.87). These findings corroborate the originally proposed three-factor solution of the PHQ–15 items [81], and provide additional support to the good reliability and validity of the scale including in Arab contexts. The Arabic PHQ-15 seems therefore to be suitable for use to assess somatic symptoms severity and screen for somatoform disorders in Arabic-speaking people.

Higher PTSD was significantly associated with more somatization. These results are confirmatory to most previous findings [35, 40, 82–87]. Somatic symptoms can coexist with PTSD and have a significant impact on the course of PTSD [40]. According to a review study of 33 studies, patients with medically unexplained physical symptoms generally report more traumatic events [82]. This was profoundly studied up to a point where PTSD became the best predictor of somatization after control for demographic variables, and other mood and anxiety disorders [35]. A study compared somatization disorder patients with PTSD to those without the syndrome. It found out that somatization disorder subjects with PTSD had more psychological distress, more interpersonal problems, and worse psychosocial functioning [83]. When evaluating specific populations of PTSD patients, such as sexually abused children, findings suggest that their PTSD symptoms were associated with somatization [84]. On another hand, torture patients who have been severely abused often show chronic symptoms of pain and dysfunction in the parts of their body where they were tortured, without any objective signs of lesion [85]. Researchers linked these somatic symptoms with feelings of hate, anger, and sadness, and these symptoms tend to become chronic [86]. Finally, several studies have been conducted on this intriguing association between somatization and PTSD, some also suggested that somatization statistically also mediates the association between peritraumatic distress and quality of life [87]. In Lebanon, millions of Lebanese are at a high-risk PTSD and a complex known as collective trauma. This risk of PTSD is increasing in the Lebanese population because of the ongoing social and economic crises, as well as the COVID-19 pandemic [62]. In the Lebanese culture, expressing PTSD symptoms through somatic complaints (e.g. bodily pain, headaches) is considered socially acceptable, since it is uncommon to express psychological distress for fear of stigmatization [88]. This explains the concurrent high prevalence of both, PTSD and somatization in this harsh alongside the severe economic crisis, growing sociopolitical unrest, and a surge of COVID-19 infections. This highlights the importance of early assessment of mental health requirements and trauma-directed psychological interventions in a country such as Lebanon [89].

Emotions are an integrated part of somatization. Understanding how emotions are experienced and how they end up being somatized is crucial in diagnosing and managing somatization. In our moderation analysis, the interaction of PTSD symptomatology from the COVID-19 pandemic by expressive suppression was significantly associated with somatization at high levels of expressive suppression. For example, emotions such as rage, are experienced as an internal energy or heat that rises from the lower abdomen to the chest, neck, and finally to the hands [90]. Diverse research has found that patients with hypertension, migraine, irritable bowel syndrome and other conditions internalize anger and thus increase their somatic problems, which has been shown to be attributable to their post-traumatic state [91]. It has also been shown that suppression of emotions, including anger, is a common finding in somatizing patients [92]. In general, patients with somatization showed higher scores on measures of regulatory strategies [93]. However, these strategies were often maladaptive as they found difficulty in expressing their feelings. Vice-versa, this suppression of negative emotions, for example, has previously also been associated with somatoform disorders [94]. Studies have suggested that ER in the form of prolonged emotional numbing or suppression reactions to trauma might also lead to somatization, as emotional disengagement can lead to an increased awareness of and focus on internal sensory perception [95]. These individuals who have trouble regulating affect might eventually try to restrain or ignore emotions, leading to the inability to describe emotions and a subsequent focus on internal sensations that could ultimately result in greater physical health complaints [96]. In the case of PTSD and the COVID-19 pandemic, it is a unique combination of ER difficulties that might predict somatization.

### Clinical implications

Our study adds to the narrow body of research revolving around the relationship between PTSD from COVID-19, and somatization in Lebanese adult residents. Findings showed the importance of emotion regulation in moderating the association between our variables. Our main finding was that relying on maladaptive emotion regulation strategies, and more particularly the tendency to use greater expressive suppression, may strengthen the likelihood of having high somatization in individuals who have high COVID-19-related PTSD symptoms. Overall, our study sheds light on the serious mental health problems endured by Lebanese residents after the COVID-19, and draws attention to the crucial need for the implementation of the appropriate preventive and coping strategies, particularly the right emotion regulation techniques. Furthermore, our results might constitute a starting point to researchers to evaluate in-depth the association between PTSD-somatization and the moderating role of ER in this relationship, whether in Lebanon or in another country. We also hope to encourage other researchers to explore other sources of PTSD, other than COVID-19, and explore other possible dependent or independent variables that could possibly be linked.

### Limitations

One of the limitations in the cross-sectional study design that was adopted in this study is the inability to prove causality. Moreover, the snowball sampling technique was used for the data collection and this method is associated with a selection bias. The elevated heterogeneity of the sample (high percentage of single participants and with a university degree) does not allow the generalizability of the results. The PCL-C scale is not validated in Lebanon. Besides, despite being relatively diversified, our relatively small sample might not be representative of the whole Lebanese population. Other traumas (e.g., participation in a car accident, injury, somatic illness, or death of close relatives, assault, rape, etc.) related to somatization, were not considered in this study, prompting a confounding bias. The study did not include any exclusion criteria and some participants with preexisting mental disorders could bias the results. The COVID-19 pandemic could not be a trauma for many people if they did not experience an infection or any difficulties during the pandemic. On the other hand, pre-pandemic PTSD was not controlled here, so it is unclear what traumas proceedings the PTSD symptoms in this study.

## Conclusion

To conclude, significant cross-sectional associations were found between PTSD and somatization in our sample of adult Lebanese residents. The moderating role of expressive suppression on the link between PTSD and somatization presents novel findings in the context of existing literature. In light of the increasing PTSD risk in the Lebanese population as a result of ongoing social and economic crises and the COVID-19 pandemic, further culturally competent research is needed to provide rigorous evidence on the prevalence, course, and severity of PTSD in Lebanon. Altogether, our findings highlight the crucial need for clinical interventions to detect, not only PTSD, but also any associated somatic symptoms, and intervene to alleviate these symptoms. Finally, in light of its apparently moderating effects, ER strategies could be effective targets of prevention and intervention in traumatized populations. Finally, longitudinal, and prospective studies are still needed to understand the interactions between PTSD, ER and post-traumatic somatization.
